# High-Sensitivity Raman Gas Probe for In Situ Multi-Component Gas Detection

**DOI:** 10.3390/s21103539

**Published:** 2021-05-19

**Authors:** Jinjia Guo, Zhao Luo, Qingsheng Liu, Dewang Yang, Hui Dong, Shuke Huang, Andong Kong, Lulu Wu

**Affiliations:** 1 College of Information Science and Engineering, Ocean University of China, Qingdao 266100, China; opticsc@ouc.edu.cn (J.G.); luozhaoqd@163.com (Z.L.); liuqingsheng@stu.ouc.edu.cn (Q.L.); kad669@163.com (A.K.); wululu1996@163.com (L.W.); 2 Laser Institute, Qilu University of Technology (Shandong Academy of Sciences), Qingdao 266000, China; 3 Institute of Machinery Manufacturing Technology, CAEP, Mianyang 621900, China; dh_caep@163.com (H.D.); huangshuke@163.com (S.H.)

**Keywords:** gas probe, in situ detection, cavity enhanced Raman spectroscopy, high sensitivity

## Abstract

Multiple reflection has been proven to be an effective method to enhance the gas detection sensitivity of Raman spectroscopy, while Raman gas probes based on the multiple reflection principle have been rarely reported on. In this paper, a multi-reflection, cavity enhanced Raman spectroscopy (CERS) probe was developed and used for in situ multi-component gas detection. Owing to signal transmission through optical fibers and the miniaturization of multi-reflection cavity, the CERS probe exhibited the advantages of in situ detection and higher detection sensitivity. Compared with the conventional, backscattering Raman layout, the CERS probe showed a better performance for the detection of weak signals with a relatively lower background. According to the 3σ criteria, the detection limits of this CERS probe for methane, hydrogen, carbon dioxide and water vapor are calculated to be 44.5 ppm, 192.9 ppm, 317.5 ppm and 0.67%, respectively. The results presented the development of this CERS probe as having great potential to provide a new method for industrial, multi-component online gas detection.

## 1. Introduction

Online gas monitoring plays an important role in industrial process control, food safety control, environmental pollution predictions and other areas [1]. Many online detection technologies have been widely applied for gas monitoring, including infrared absorption spectroscopy [2,3], gas chromatography (GC) [4,5], mass spectrometry (MS) [5,6], laser Raman spectroscopy [7,8], etc. Among them, infrared absorption technology has the advantages of relatively high detection sensitivity and flexible operations but usually focuses on one gas and cannot detect some infrared inactive gases, e.g., N_2_, O_2_ and H_2_ [9]. The GC, MS and GC-MS methods are considered the most widespread analytical procedures because of their superior sensitivity and selectivity. However, these methods are time-consuming, expensive and require tedious sample pretreatment procedures [10,11], which might restrict their wide application as online analyzers in the industrial field. Owing to its relatively low sensitivity, laser Raman spectroscopy has rarely been used in gas monitoring [12]. Nevertheless, the innovation of photoelectric technology widely promoted the development of laser Raman spectroscopic technology, making it a broader research prospect [13].

Compared with infrared absorption technology, the most significant advantage of Raman spectroscopy is its in situ, multi-component measurement ability [14]. Moreover, with its simple sample pretreatment process and low maintenance cost, it is an attractive option for online gas analysis in harsh factory environments. In addition, Raman spectroscopy has the advantage of multicomponent detection, such as hydrocarbon gases (CH_4_ and C_2_H_4_), O_2_, N_2_, H_2_, SO_2_, H_2_S, etc. Considering the limitations of low detection sensitivity [15,16,17], many studies have been involved in approaches to improve the detection sensitivity of Raman spectroscopy. Previous studies have indicated that multi-reflection cavity was an effective method to improve the sensitivity of Raman spectroscopy in gas detection [10,11]. Atmosphere Recovery Inc. invented a laser Raman gas analyzer with a combination of a laser resonant cavity, sample cell and an array of eight signal collection channels on both sides, which achieved the combination of filter and photoelectric sensors and was capable of detecting one single gas species [18]. Shanghai Jiao Tong University developed a near confocal cavity [19,20] and Chongqing University [21] invented a V-shaped cavity to enhance the Raman signal intensity. The Institute of Monitoring of Clinical and Ecological Systems in Russia improved Raman signal intensity by increasing the sample pressure [22]. The University of Jena in Germany developed a fiber enhanced Raman spectroscopy by using the hollow core photonic crystal fiber to improve detection sensitivity [23,24,25]. The University of Sheffield designed optical feedback CW diode lasers to enhance the Raman signal [26], in which the feedback technology improved the stability of the Raman signal. The development of all these technologies has greatly promoted the application of laser Raman spectroscopy in the online monitoring of industrial gas. For further applications, miniaturization as well as probe-based and simultaneous multi-point detection would be the new direction of requirements. At present, many companies have commercial products of laser Raman probes, such as the Ocean Optics Raman probe RPB series [27] and probe RPB-532-N-FS series of the Shanghai Oceanhood Photoelectric Technology Company [28], but most of these products are mainly designed for liquid and solid samples. Based on our understanding, Kaiser Optical Systems (USA) released a gas probe named “Kaiser Raman Airhead Probe” with a mirror added into the front of a conventional backscattering system, generating a theoretically four-fold intensified Raman signal [29].

As mentioned above, the multi-reflection, cavity enhanced Raman spectroscopy (CERS) system has a high sensitivity for gas detection, but so far, few Raman gas probes with a CERS system have been used for industrial gas detection [30]. This paper proposes the development of a set of gas Raman probes based on CERS. The detection ability of this probe was evaluated by measuring standard gas and compared to conventional, commercial probes. The results show the ability and potential of the prepared CERS probe in industrial gas monitoring.

## 2. Experimental Setup

### 2.1. Experimental System

**Figure 1:**
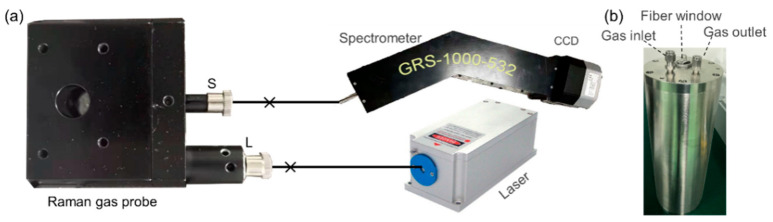
Diagram of the Raman probe experimental system showing (**a**) the optical setup and (**b**) the sealed stainless-steel chamber.

An experimental setup was used to achieve remote monitoring of gas components in special environments, as shown in Figure 1 of this paper. The multi-pass cavity was integrated into one probe within a size of 80 × 60 × 40 mm. Two ventilation holes on the top and bottom of the probe surface were directly connected to the multi-reflection cavity. Additionally, there were two SMA905 fiber adapters mounted on the front surface of the probe. A diode pumped the 532 nm laser with an average power of 2.5 W, which was used as the exciting source. It was coupled into a 50 μm optical fiber, with output power through the fiber of 1.6 W with a coupling efficiency of 64%. Then, the optical fiber was connected to the probe via a a SMA905 fiber optic connector, which is marked as “L” in Figure 1a. After being collimated and beam compressed, the incident light entered the multi-reflection cavity. The generated Raman signal was collected by achromatic doublets and conducted into a GRS-1000-532 spectrometer (spectral range of 0–5000 cm^−1^, resolution 10 cm^−1^, manufactured by Qingdao Jinpusheng Tech Company) through another connector (marked as “S”) and a 600 μm optical fiber. A sealed stainless-steel chamber with a size of φ200 (i.d.) × 300 mm and a thickness of 15 mm was used for experiments. In the experimental process, the Raman probe was housed in the sealed stainless-steel chamber, as shown in Figure 1b. There were three ports on the top cover of the chamber, two of which were ventilation holes for filling the chamber with sample gas, and the third one was a fiber-optic feed through the port. Two optical fibers connected the probe and spectrometer, passing the top cover of the chamber through the fiber-optic port.

### 2.2. Design of the Cavity Enhanced Raman Spectroscopy (CERS) Gas Probe

**Figure 2:**
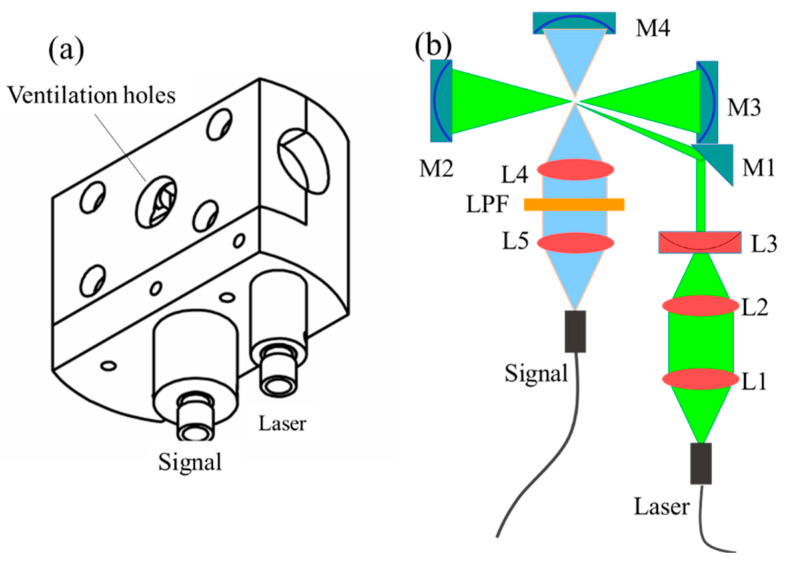
Schematic of the multi-reflection cavity probe design. (**a**) Structure drawing of high-sensitivity Raman gas probe; (**b**) the schematic of high-sensitivity Raman gas probe.

The main structure of the CERS gas probe is shown in Figure 2. It includes three parts: the excitation light collimation assembly, multi-pass cavity and signal collection assembly. The detail structure is shown in Figure 2b, where the excitation light was collimated and compressed through lenses L1, L2 and L3 and then reflected into the nearby concentric cavity. The cavity was composed of two concave mirrors (M2 and M3) with a focal length of 10 mm and a diameter of 12 mm, with the distance between M2 and M3 being approximately 40 mm. The laser oscillated in the cavity and excited the gas molecules in the cavity to generate the Raman signal, which was collected by signal collection lenses (L4 and L5) and coupled into an optical fiber with a core diameter of 600 μm. A long pass filter (LPF) was then mounted between the two lenses. A concave mirror (M4) was placed on the opposite side of the signal collection assembly to increase the solid angle collection. Two ventilation holes on the top and bottom of the probe enabled gas exchange between the inside and outside of the multi-reflection cavity.

### 2.3. Measurements

Standard gas samples of CH_4_, H_2_ CO_2_, O_2_ and N_2_ were purchased from Yantai Deyi Gas Co. Ltd., China. To recognize the Raman spectra peaks of CH_4_, H_2_, CO_2_, O_2_ and N_2_, their standards were mixed to obtain the target concentrations (5%, 1%, 5%, 1% and 88%, volume ratios) and detected using this CERS gas probe. The integration time used in this experiment was 10 s, the number of accumulations was 10, and 9 sets of spectra were acquired for each measurement. The exposition time was 900 s for each sample. The ambient temperature was 25 degrees. In order to ensure the accuracy of the measurements, the chamber was flushed 3 times using target gas before testing, and then the pressure in the chamber was maintained at one bar.

In order to investigate the detection capability of the CERS probe for gases, different concentrations of gas samples were prepared using N_2_ as supplements. Standard CH_4_ gas was mixed to obtain a series of concentrations (2076, 10,934, 50,000 and 10,000 ppm), and standard H_2_ and CO_2_ gases were also prepared to different concentrations (1986, 5050, 10,020 and 39,900 ppm; 1996, 10200, 50,200 and 80,030 ppm). During the testing process, the relative humidity recorded by the humidity sensor was 11.5%, 18.7%, 26.2%, 35.2% and 50.0%, respectively. Furthermore, in order to further investigate the application performance of this CERS probe, a combined standard gas containing 2000 ppm of CO_2_, 1% O_2_ and N_2_ was prepared and then detected by this CERS probe and a commercial backscattering Raman probe, respectively. Their Raman spectra of the combined standard gases were collected and analyzed for further cooperation.

## 3. Results and Discussion

**Figure 3:**
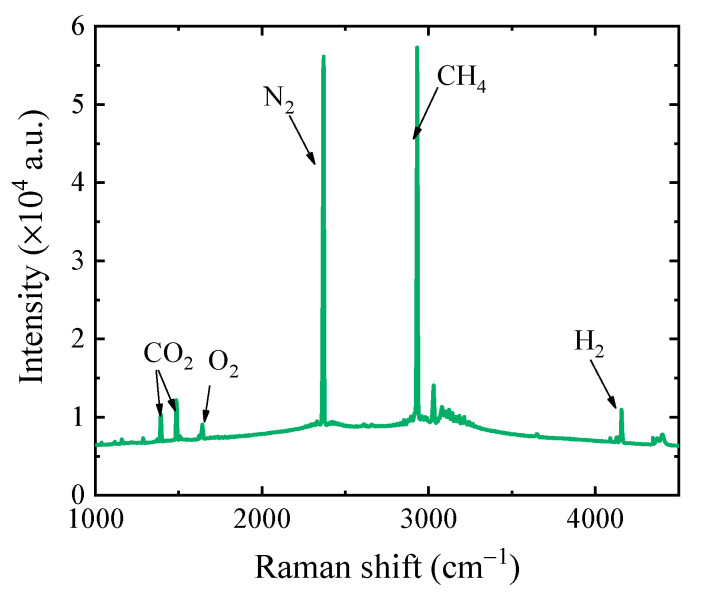
A typical detection result detected with the cavity enhanced Raman spectroscopy (CERS) probe.

Figure 3 shows that the typical combinations of O_2_, CO_2_, CH_4_, H_2_ and N_2_ gas samples were detected using this CERS gas probe. The Raman peaks of various gas components could obviously be easily distinguished, such as N_2_ at 2331 cm^−1^, O_2_ at 1555 cm^−1^, CO_2_ at 1289 cm^−1^ and 1387 cm^−1^, H_2_ at 4156 cm^−1^ and CH_4_ at 2917 cm^−1^, as shown in Figure 3.

**Figure 4:**
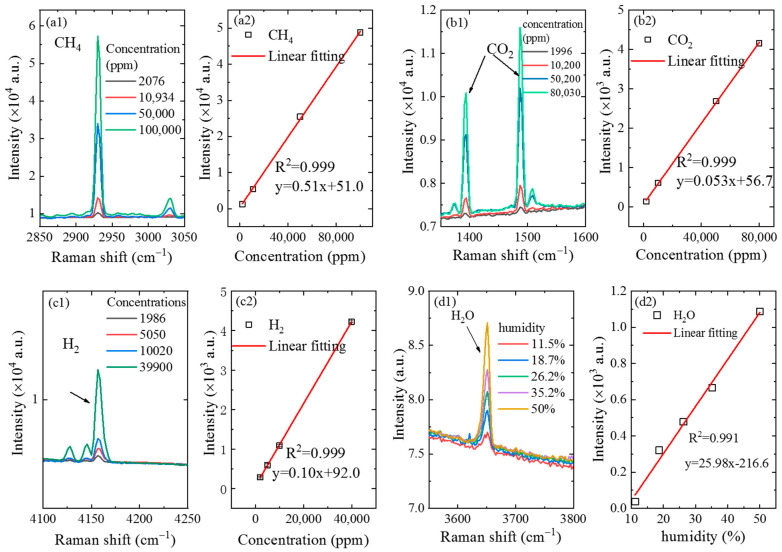
Raman spectra and calibration curve of gas samples in different concentrations obtained by the CERS probe. (**a1**) Raman spectra of CH_4_; (**a2**) calibration curve of CH_4_ based on the peak intensity with Raman shift of 2917 cm^−1^; (**b1**) Raman spectra of H_2_; (**b2**) calibration curve of H_2_ based on the peak intensity with Raman shift of 4156 cm^−1^; (**c1**) Raman spectra of CO_2_; (**c2**) calibration curve of CO_2_ based on the peak intensity with Raman shift of 1387 cm^−1^; (**d1**) Raman spectra of water vapor; (**d2**) calibration curve of H_2_O based on the peak intensity with Raman shift of 3650 cm^−1^.

In order to evaluate the detecting capability of the CERS probe for gases, different concentrations of gases were detected by the CERS probe to retrieve the calibration curve and detection sensitivity. The Raman spectra of CH_4_ gas on four different concentration levels were collected, as shown in Figure 4a1. After gases were filled into the sealed chamber to displace the air inside, the pressure was reduced to 1 atm and the probe was put into the sealed chamber to detect the gas concentration. It can be observed from Figure 4a1 that the Raman signal intensity of CH_4_ gas increased with the increase of the concentration of CH_4_ gas. Based on the peak intensities of the gases on different concentration levels, the relationship between peak intensities and concentrations was established. As shown in Figure 4a2, there was a good linearity with correlation coefficient R^2^ = 0.999 [31]. Based on the calculation formula for the limit of detection (LOD), LOD = 3σ/s (where σ is the noise intensity and s is the slope of the calibration curve) [32], the LOD was 44.5 ppm for CH_4_, with a noise intensity (σ) of 7.57, a slope (S) of 0.51 and relative standard deviations of 2.2%. Noise intensity was acquired by calculating the standard deviation from the data without Raman peaks (3700–3900 cm^−1^).

The Raman spectra of CO_2_ gas on different concentration levels obtained by the CERS probe are shown in Figure 4b1. It can be seen from the figure that CO_2_ has two strong Raman peaks, which are at 1289 cm^−1^ and 1387 cm^−1^, respectively. In order to improve the accuracy of this analytical method, the intensity of the latter Raman peak was chosen for quantitative calibration, due to the higher intensity than that of the former one. It can also be observed that the Raman signal intensity of CO_2_ gas increased with the increase of the concentration, indicating that higher contents of gas correspond to high signal intensity values. Furthermore, the relationship between peak intensity and concentration was established as presented in Figure 4b2, showing that the Raman signal of CO_2_ gas at 1387 cm^−1^ had a good linearity with a correlation coefficient R^2^ = 0.999. Based on the 3σ criterion, the LOD of this probe was calculated to be at 317.5 ppm for CO_2_ gas detection, with relative standard deviations of 3.2%.

With this same method, the Raman signals of H_2_ gas on different concentration levels were obtained, as shown in Figure 4c1. It can be observed that the Raman peak of H_2_ was located at 4156 cm^−1^, and the calibration curve had a good linear with the correlation coefficient R^2^ = 0.999. The calculated LOD for H_2_ gas was shown to arrive at 192.9 ppm with relative standard deviations of 2.5%. The error bar of peak intensity was approximately 24, which was too small to display clearly in Figure 4a2,b2,c2).

Significantly, the CERS probe could also be used to detect the concentration of water vapor in the air. During the experiment, the relative humidity of the air was at approximately 50%. The miniaturized temperature–humidity sensor and the CERS probe were put into the sealed chamber at the same time. Air with a humidity of 50% was firstly filled into the sealed chamber. Next, dry sample gas was gradually filled into the sealed chamber to dilute the water vapor, and the pressure inside the sealed chamber was kept at 1 atm. During the experimental procedure, the humidity of the gas inside the chamber was measured using both a Raman probe and the temperature–humidity sensor. The experimental results are exhibited and compared in Figure 4d1, where the relative humidity obtained by the humidity sensor is 11.5%, 18.7%, 26.2%, 35.2% and 50.0%, respectively. The obtained Raman signals of water vapor, as shown in Figure 4d1,d2, exhibit a good linear relationship between Raman signal intensity and humidity, with an R^2^ of 0.991, an LOD of 0.7% for humidity and low relative standard deviations of 2.0%.

**Figure 5:**
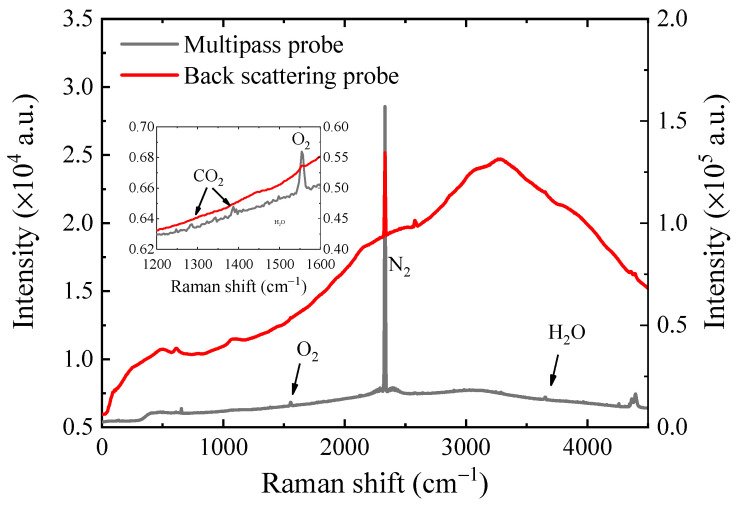
Result comparison of CERS probe and commercial backscattering Raman probe for 2000 ppm CO_2_ and 1% O_2_.

In order to further investigate the performance of this multiple reflection CERS probe, these experiment results obtained from the CERS Raman probe and a commercial backscattering Raman probe were compared by measuring a combined standard gas sample containing 2000 ppm CO_2_, 1% O_2_ and N_2_. The experimental results are shown in Figure 5, where the black line represents the results of the multiple reflection CERS probe and the red line represents the results of the commercial backscattering Raman probe. It can be seen from Figure 5 that the spectra collected by the CERS probe had a relatively lower background, and therefore, the weaker Raman peaks of CO_2_, O_2_ and H_2_O could be more easily revealed. In contrast, the commercial backscattering Raman probe had a higher absolute intensity of the N_2_ peak and higher background intensity, making the weaker peaks of CO_2_, O_2_ and H_2_O invisible. As shown in Figure 5, we could find that the signal-to-background ratio of the multiple reflection CERS probe was around 4.6 times higher than that of the commercial backscattering Raman probe. Therefore, it could be deduced that the homemade multiple reflection CERS probe had a better signal-to-background ratio than the commercial probe, which indicates that the multiple reflection CERS probe would have better sensitivity.

## 4. Conclusions

For industrial online gas detection, a set of gas Raman probes based on CERS have been developed, which are small in volume and connected to the trunk of the instrument containing the laser and the spectrometer with two optical fibers. The multi-reflection CERS probe has been proven to be an effective method to improve sensitivity for in situ, multi-component gas detection. With the aid of the multi-reflection cavity, the LODs of CH_4_, CO_2_ and H_2_ were at 44.5, 317.5 and 192.9 ppm, respectively. In addition, the probe can also measure the concentration of water vapor with a detection limit of 0.7% relative humidity. Furthermore, compared with a conventional backscattering Raman probe, the CERS probe could be more favorable for the detection of weak signals, exhibiting a lower background and better signal/background ratio. The results showed the great ability and potential of the new CERS probe in industrial gas monitoring.

## Data Availability

Data sharing not applicable.
